# Validation of the CONSULT-PSYCHIATR Scale: reasons for reluctance to seek psychiatric consultation among workers in Peru

**DOI:** 10.3389/fpsyt.2026.1836597

**Published:** 2026-07-08

**Authors:** Christian R. Mejia, Gianella Vera, Oscar Mamani, Victor Serna-Alarcón, Jaime A. Yáñez, Neal M. Davies, María J. Erazo-Muñoz, Leonel Vega-Pérez, Camilo Vega-Useche

**Affiliations:** 1 Asociación Médica de Investigación y Servicios en Salud, Lima, Peru; 2 Sociedad Científica de Estudiantes de Medicina de la Universidad Continental, Huancayo, Peru; 3 Facultad de Ciencias de la Salud, Universidad Señor de Sipán, Chiclayo, Peru; 4 Universidad Privada Antenor Orrego, Piura, Peru; 5 Hospital Regional José Cayetano Heredia, EsSalud, Piura, Peru; 6 Escuela de Posgrado, Universidad Internacional Iberoamericana, Campeche, Mexico; 7 University of Alberta, Edmonton, AB, Canada; 8 Universidad Pedagógica y Tecnológica de Colombia, Tunja, Colombia; 9 Departamento de Medicina Crítica y Cuidado Intensivo, Fundación Santa Fe de Bogotá, Bogotá, Colombia

**Keywords:** help-seeking, mental health, Peru, psychiatrist, validation, workers

## Abstract

**Introduction:**

Workers may be reluctant to attend psychiatric consultation because of stigma, interpersonal concerns, and practical barriers. In Peru, brief instruments specifically designed to assess these reasons in working populations are limited. This study examined the content validity, internal structure, and internal consistency of a Peruvian scale assessing reasons why workers are reluctant to attend consultations with a psychiatrist.

**Methods:**

An instrumental study was conducted in Peru. Item development was informed by a literature review and expert input. An initial 10-item version was evaluated by 20 experts for relevance, representativeness, and clarity using Aiken’s V. After a pilot test, the questionnaire was administered to 3001 workers from several Peruvian departments using non-random sampling. Item distribution was examined using descriptive statistics, skewness, excess kurtosis, floor and ceiling effects, and constant-response patterns. The sample was randomly divided into exploratory and confirmatory subsamples. A polychoric exploratory factor analysis was conducted in the exploratory subsample, followed by confirmatory analyses in the independent confirmatory subsample. Internal consistency was estimated using Cronbach’s alpha, ordinal alpha, and ordinal omega total.

**Results:**

Most Aiken’s V coefficients were 0.70 or higher, with only one lower value for the representativeness of item 9. The 10-item version showed adequate item distributions, although 46.95% of participants provided constant responses across all items, prompting sensitivity analyses. Exploratory factor analysis supported a one-factor structure, explaining 80.96% of the variance, with factor loadings ranging from 0.8425 to 0.9365. This structure remained stable after excluding constant-response patterns. In the confirmatory subsample, the 10-item one-factor model showed high standardized loadings and favorable CFI, TLI, and SRMR values, although RMSEA was less favorable and was interpreted jointly with the remaining indices. Reliability estimates were high in both the main and sensitivity analyses.

**Discussion:**

The CONSULT-PSYCHIATR is a brief 10-item instrument with evidence of content validity, internal structure, and internal consistency for assessing perceived reasons why workers in Peru may be reluctant to attend psychiatric consultation. Although the findings are encouraging, further studies are needed to examine its performance in other populations and settings and to assess additional sources of validity evidence.

## 1 Introduction

Mental disorders are a leading cause of disability worldwide, and a substantial proportion of people with clinically relevant symptoms do not receive timely or adequate care (1). In the Americas, the mental health treatment gap remains considerable, particularly in Latin America, contributing to persistent unmet need (2). Evidence from large international surveys shows that, beyond service availability, help-seeking is strongly shaped by low perceived need and attitudinal barriers, often more than by structural barriers (3). Consistent findings have been reported in World Mental Health data from countries in the Americas, where low perceived need is common among those not using services and attitudinal reasons remain frequent among those who do perceive need (4).

Stigma is a central driver of reluctance to seek professional help. Systematic reviews indicate that mental health-related stigma has a small-to-moderate negative effect on help-seeking, and disclosure concerns are repeatedly reported as key stigma-related barriers (5). Meta-analytic evidence further supports an association between stigma and reduced active help-seeking (6). Other commonly reported barriers include embarrassment, limited mental health literacy, and preference for self-reliance, as summarized in systematic evidence on help-seeking barriers (7). Together, this literature suggests that reluctance to seek psychiatric care is shaped not only by the availability of services, but also by how psychiatric consultation is socially interpreted and personally anticipated.

In working populations, reluctance to seek mental health care may be amplified by workplace-specific concerns. Employees may fear negative professional consequences, prejudice, or confidentiality breaches if they disclose emotional difficulties or seek specialized care (8). At the same time, workplaces are increasingly used as platforms for mental health promotion and early intervention. Evidence syntheses identify potentially beneficial workplace interventions for common mental disorders, although effects vary by intervention type and implementation (9). More recent evidence suggests that screening alone is unlikely to improve outcomes unless it is linked to facilitated access to appropriate interventions, and confidentiality remains a critical consideration (10). In this context, identifying modifiable reasons why workers may be reluctant to attend psychiatric consultation is especially relevant.

In Peru, mental health reforms have expanded community-based services and promoted a shift from psychiatric hospitals toward community mental health centers (11, 12). However, greater service availability does not fully explain why employed adults may still avoid psychiatric consultation. Existing instruments assess related but distinct constructs, such as self-stigma associated with seeking psychological help, broad barriers to accessing mental health care, or general attitudes toward professional psychological help (13–15). Although these measures are valuable, they were not designed specifically to assess concrete reasons for reluctance to attend psychiatric consultations among workers in Peru. A brief, context-appropriate instrument focused on this decision may therefore be useful for occupational health research and workplace mental health initiatives aimed at identifying and addressing modifiable barriers to care.

Therefore, the aim of this study was to examine the content validity, internal structure, and internal consistency of a Peruvian scale assessing reasons why workers are reluctant to attend consultations with a psychiatrist.

## 2 Materials and methods

### 2.1 Research design

This instrumental study was conducted in Peru to develop and evaluate the psychometric properties of the CONSULT-PSYCHIATR scale, an instrument designed to assess reasons why workers may be reluctant to attend psychiatric consultation. The study followed sequential stages of item development, expert-based content validation, pilot testing, field administration, and psychometric evaluation of the internal structure and reliability of the instrument. Data were collected from workers residing in several Peruvian departments, including Lima, Huancayo, Arequipa, La Libertad, and Piura. Participants were recruited through non-probabilistic procedures, using occupational health contacts and snowball dissemination strategies. The final analytic sample comprised 3001 workers who completed the 10-item version of the scale.

### 2.2 Validation steps

A literature search was conducted using Scopus, PubMed, SciELO, and Web of Science databases to identify previously reported reasons for reluctance to seek psychiatric or mental health care, with particular attention to attitudinal, interpersonal, and practical barriers relevant to the intended construct. This review informed the initial conceptual domains and the wording of candidate items. In parallel, a group of professionals with experience in mental health, occupational health, and worker-related contexts, including psychologists, psychiatrists, occupational physicians, and occupational psychologists, provided qualitative input on the conceptual relevance, clarity, and contextual appropriateness of the proposed content. Based on this combined process, a preliminary list of fifteen candidate items was generated. Five items were subsequently removed after qualitative review because they were considered redundant, insufficiently specific to the intended construct, or less suitable for the Peruvian worker context.

The resulting 10-item version was then subjected to formal expert judgment. Twenty experts, including occupational physicians, psychologists, psychiatrists, and professionals with experience in organizational or leadership settings, evaluated each item in terms of relevance, representativeness, and clarity. Expert ratings were summarized through descriptive statistics and Aiken’s V coefficients with 95% confidence intervals, as detailed in the statistical analysis section.

Subsequently, a pilot test was conducted to assess comprehension, acceptability, and approximate completion time. Participants completed the instrument in approximately 6 minutes. No major problems in item comprehension or survey flow were identified; only minor questions related to the meaning of specific terms or general response instructions were reported. Following this stage, the 10-item questionnaire was retained for field administration.

Using snowball sampling, occupational physicians and other leaders from occupational health companies were contacted. These contacts supported dissemination of the survey invitation to workers, but they did not have access to individual responses or participation records. The invitation explained the objective of the study, the anonymous nature of data collection, the voluntary character of participation, the right to decline any question, and the right to discontinue participation at any time without consequences. The final survey included 3001 workers. Because recruitment relied on open dissemination through occupational health networks and snowball procedures, the total number of workers who received the invitation could not be determined; therefore, a conventional response rate could not be calculated. After these stages were completed, the data analysis was carried out. The instrument was named the CONSULT-PSYCHIATR scale.

The CONSULT-PSYCHIATR was originally developed, administered, and psychometrically evaluated in Spanish. To preserve the wording of the validated instrument while facilitating readability in this English-language manuscript, the final items are presented in both their original Spanish version and an English rendering in the main text.

### 2.3 Data analysis

Expert ratings were summarized using means, standard deviations, Aiken’s V coefficients, and their 95% confidence intervals. For the field application, item performance was first examined through means, standard deviations, skewness, excess kurtosis, and the observed response range. Item-level floor and ceiling effects were also calculated as the proportion of participants selecting the minimum and maximum response categories, respectively. In addition, because a substantial proportion of participants provided identical responses across all 10 items, the frequency of constant response patterns was quantified and incorporated into sensitivity analyses.

To reduce capitalization on chance and enable internal cross-validation of the factorial structure, the total sample was randomly divided into two independent subsamples: an exploratory subsample for factor discovery (n = 1500) and a confirmatory subsample for model testing (n = 1501). In the exploratory subsample, the suitability of the data for factor analysis was assessed using the Kaiser–Meyer–Olkin (KMO) measure and Bartlett’s test of sphericity. Given the ordered categorical nature of the items, the primary exploratory factor analysis was conducted on a polychoric correlation matrix. The dimensional structure was evaluated through the eigenvalue distribution, scree plot, proportion of variance accounted for by the first factor, and the magnitude of factor loadings. As a robustness analysis, the same polychoric exploratory factor analysis was repeated after excluding participants who provided constant responses across the 10 items.

The hypothesized one-factor structure was then tested in the confirmatory subsample. First, a classical confirmatory factor analysis was estimated using structural equation modeling, and standardized factor loadings and item-level explained variance were reported. Model fit was assessed using the chi-square statistic, degrees of freedom, Comparative Fit Index (CFI), Tucker–Lewis Index (TLI), Root Mean Square Error of Approximation (RMSEA) with its 90% confidence interval, and Standardized Root Mean Square Residual (SRMR). Consistent with conventional guidance, CFI and TLI values ≥ 0.90 and SRMR values ≤ 0.08 were interpreted as supportive of acceptable fit, while RMSEA was interpreted jointly with the remaining indices rather than in isolation (16). Modification indices were inspected only for diagnostic purposes and were not used to perform *post hoc* item deletion or data-driven model re-specification.

Because the indicators were ordinal, an additional confirmatory analysis was conducted using generalized structural equation modeling with an ordered probit link. This ordinal model was treated as a complementary evaluation of the proposed latent structure, and Akaike and Bayesian information criteria were reported. A sensitivity version of the ordinal confirmatory model was also estimated after excluding participants with constant response patterns across all items.

Internal consistency was first estimated using Cronbach’s alpha in the total sample and in the sensitivity sample excluding constant responses. To provide reliability evidence more aligned with ordinal response scales, ordinal alpha and ordinal total omega were additionally estimated from the polychoric correlation matrices in the exploratory subsample, both for the complete exploratory analysis and for the sensitivity analysis excluding constant response patterns. All statistical analyses were conducted in Stata, version 19 (StataCorp, College Station, TX, USA).

### 2.4 Ethics

The study protocol was reviewed and approved by the Comité de Bioética of the Universidad Privada Antenor Orrego, Peru, under Resolution No. 0063-2023-UPAO, issued on 8 March 2023. The approved protocol was titled *“Validación y aplicación de escalas para evaluar la salud mental en trabajadores.”* The study was conducted in accordance with the ethical principles for medical research involving human participants set forth in the Declaration of Helsinki. Participation was voluntary and anonymous. Before accessing the questionnaire, all potential participants were informed about the purpose of the study, the anonymous nature of data collection, their freedom to answer or decline any question, and their right to discontinue participation at any time without consequences. Electronic informed consent was obtained through an explicit acceptance button or checkbox before survey completion.

Given that recruitment was facilitated through occupational physicians and contacts linked to occupational health settings, participation was presented as entirely voluntary and independent of any employment-related obligation. Employers, supervisors, and recruiting intermediaries did not have access to individual responses. Data were analyzed and reported only in aggregate form, with no personally identifiable information disclosed.

## 3 Results

### 3.1 Content validity based on expert judgment

The initial 10-item version of the CONSULT-PSYCHIATR scale was evaluated by 20 experts in terms of relevance, representativeness, and clarity. As shown in **Table 1**, Aiken’s V coefficients were generally favorable across the three domains. Relevance and clarity coefficients were 0.70 or higher for all items, while representativeness also reached values of 0.70 or higher in nine of the ten items. The only lower estimate was observed for the representativeness of Item 9 (*V* = 0.60; 95% CI: 0.40–0.80). Given that this item met acceptable values for relevance and clarity and was considered conceptually pertinent during the content review process, it was retained for subsequent psychometric evaluation.

**Table 1:** Content validity of the initial 10-item CONSULT-PSYCHIATR scale based on Aiken’s V.

Item	Relevance (n = 20)				Representativeness (n = 20)				Clarity (n = 20)			
	M	SD	V	95% CI	M	SD	V	95% CI	M	SD	V	95% CI
Item 1	2.7	0.5	0.9	0.7–1.0	2.8	0.4	0.9	0.8–1.0	2.7	0.6	0.9	0.7–1.0
Item 2	2.3	0.9	0.8	0.6–0.9	2.3	0.9	0.8	0.6–0.9	2.4	0.8	0.8	0.6–0.9
Item 3	2.4	0.8	0.8	0.6–0.9	2.4	0.8	0.8	0.6–0.9	2.6	0.7	0.9	0.7–0.9
Item 4	2.4	0.8	0.8	0.6–0.9	2.5	0.7	0.8	0.7–0.9	2.5	0.8	0.8	0.7–0.9
Item 5	2.4	0.8	0.8	0.6–0.9	2.6	0.7	0.9	0.7–0.9	2.7	0.6	0.9	0.7–1.0
Item 6	2.5	0.7	0.8	0.7–0.9	2.4	0.7	0.8	0.6–0.9	2.6	0.6	0.9	0.7–1.0
Item 7	2.4	0.8	0.8	0.6–0.9	2.3	0.7	0.8	0.6–0.9	2.5	0.7	0.8	0.7–0.9
Item 8	2.3	0.9	0.8	0.6–0.9	2.2	0.9	0.7	0.6–0.9	2.4	0.9	0.8	0.6–0.9
Item 9	2.0	1.1	0.7	0.5–0.8	1.9	1.0	0.6	0.4–0.8	2.4	0.9	0.8	0.6–0.9
Item 10	2.0	1.1	0.7	0.5–0.8	2.1	1.2	0.7	0.5–0.8	2.1	1.1	0.7	0.5–0.8

M, mean; SD, standard deviation; V, Aiken’s V; CI, confidence interval.

### 3.2 Item distribution and response-pattern assessment

Descriptive statistics and distributional properties of the 10 items are presented in **Table 2**. Item means ranged from 1.362 to 1.668, and standard deviations ranged from 1.058 to 1.194. Skewness values were low and positive across all items, while excess kurtosis values were negative but remained within an acceptable descriptive range, suggesting no extreme distributional distortions at the item level. Floor effects ranged from 17.93% to 24.89%, whereas ceiling effects were consistently low, ranging from 3.07% to 6.96%. These findings indicate a greater concentration of responses in the lower end of the scale, without evidence of meaningful ceiling accumulation. Given the unusually high degree of similarity across item responses identified during data auditing, constant-response patterns were specifically examined. A total of 1409 participants (46.95%) selected the same response category across all 10 items, while 1592 participants (53.05%) showed at least one response variation across the scale (**Supplementary Table 1**). This finding motivated sensitivity analyses excluding constant-response patterns in the exploratory, confirmatory, and reliability assessments.

**Table 2:** Descriptive statistics, distributional properties, and floor/ceiling effects of the 10 CONSULT-PSYCHIATR items.

Item	Mean	SD	Skewness	Excess kurtosis	Floor effect (%)	Ceiling effect (%)
Item 1	1.668	1.194	0.198	−0.895	20.09	6.96
Item 2	1.491	1.078	0.302	−0.586	20.76	3.77
Item 3	1.603	1.122	0.212	−0.753	19.23	4.80
Item 4	1.392	1.079	0.361	−0.569	24.79	3.33
Item 5	1.435	1.075	0.316	−0.618	22.89	3.20
Item 6	1.481	1.094	0.286	−0.694	22.06	3.53
Item 7	1.454	1.082	0.327	−0.605	22.19	3.53
Item 8	1.660	1.135	0.191	−0.761	17.93	5.70
Item 9	1.362	1.058	0.383	−0.493	24.89	3.07
Item 10	1.586	1.167	0.267	−0.744	21.73	6.36

N = 3001 for all items. Floor effect corresponds to the percentage of responses in the lowest response category; ceiling effect corresponds to the percentage of responses in the highest response category.

### 3.3 Exploratory internal structure in the exploratory subsample

The total sample was randomly divided into two independent subsamples. The exploratory factor analysis was conducted in the first subsample (n = 1500). Using a polychoric correlation matrix, the data showed excellent factorability, with a Kaiser–Meyer–Olkin index of 0.965 and a statistically significant Bartlett’s test of sphericity: χ²(45) = 19072.405, *p* < 0.001. The exploratory analysis supported a dominant one-factor solution for the 10-item scale. The first factor had an eigenvalue of 8.096 and accounted for 80.96% of the total variance. As shown in **Table 3**, all factor loadings were high, ranging from 0.8425 to 0.9365, with correspondingly low uniqueness values.

**Table 3:** Exploratory factor analysis of the 10-item CONSULT-PSYCHIATR scale using polychoric correlations.

Item	Polychoric EFA, full exploratory subsample (n = 1500)		Polychoric EFA excluding constant-response patterns (n = 789)	
	Loading	Uniqueness	Loading	Uniqueness
Item 1	0.8425	0.2902	0.6734	0.5466
Item 2	0.9192	0.1550	0.8307	0.3099
Item 3	0.8799	0.2258	0.7446	0.4455
Item 4	0.9179	0.1575	0.8532	0.2720
Item 5	0.9321	0.1312	0.8771	0.2306
Item 6	0.9365	0.1230	0.8806	0.2246
Item 7	0.9312	0.1328	0.8683	0.2460
Item 8	0.8713	0.2409	0.7192	0.4828
Item 9	0.8999	0.1902	0.8120	0.3407
Item 10	0.8620	0.2570	0.7153	0.4883

Full EFA: first eigenvalue = 8.096; explained variance = 80.96%; KMO = 0.965; Bartlett’s χ²(45) = 19072.405, p < 0.001. Sensitivity EFA: first eigenvalue = 6.413; explained variance = 64.13%; KMO = 0.945; Bartlett’s χ²(45) = 5699.721, p < 0.001.

A sensitivity analysis was then performed after excluding participants with constant-response patterns, leaving 789 participants in the exploratory subsample. The one-factor structure remained stable. The polychoric matrix continued to show strong adequacy for factor analysis (KMO = 0.945; Bartlett’s χ²(45) = 5699.721, *p* < 0.001). The first factor had an eigenvalue of 6.413 and explained 64.13% of the variance. Factor loadings remained substantial, ranging from 0.6734 to 0.8806 (**Table 3**). Thus, although the magnitude of the associations decreased after removing constant-response patterns, the unidimensional structure was preserved.

### 3.4 Confirmatory internal structure in the independent confirmatory subsample

**Figure 1:**
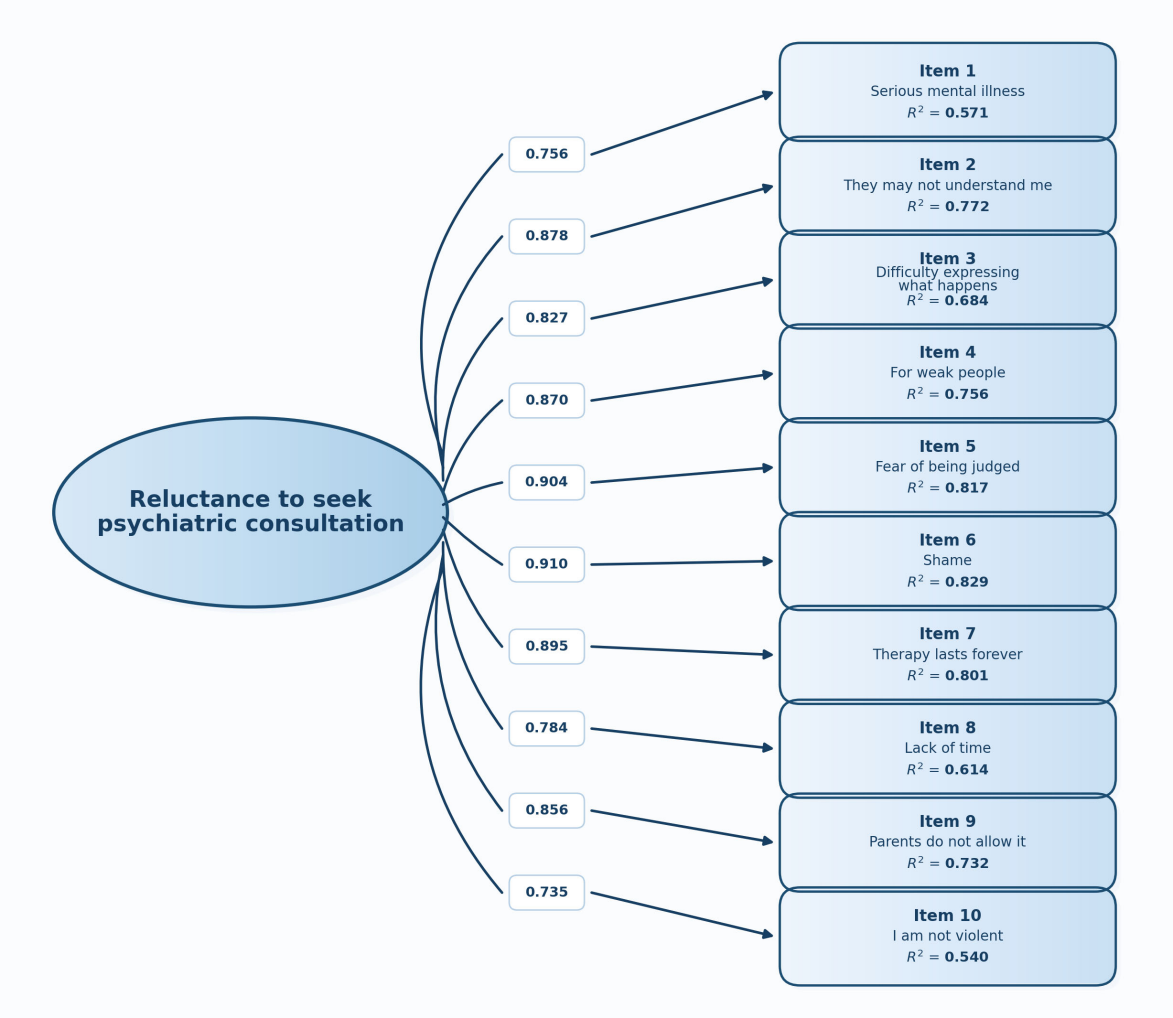
Confirmatory factor analysis of the 10-item CONSULT-PSYCHIATR scale in the independent confirmatory subsample. The figure shows the standardized factor loadings for the one-factor model. All items loaded positively on the latent construct representing reasons for reluctance to attend psychiatric consultation. Standardized factor loadings ranged from 0.7351 to 0.9104.

The hypothesized one-factor model was tested in the independent confirmatory subsample (n = 1501). The classical CFA showed high standardized factor loadings for all 10 items, ranging from 0.7351 to 0.9104, with item-level explained variance values ranging from R² = 0.5403 to R² = 0.8288 (**Table 4**; **Figure 1**). The overall fit of the 10-item one-factor model was favorable according to the incremental and residual-based indices: CFI = 0.972, TLI = 0.964, and SRMR = 0.022. The RMSEA was 0.090 with a 90% confidence interval of 0.082–0.097, indicating a less favorable absolute fit estimate that should be interpreted jointly with the remaining indices rather than in isolation (**Table 5A**). No *post hoc* item deletions or data-driven model re-specifications were applied. Modification indices were examined only as diagnostic information.

**Table 4:** Confirmatory factor analysis of the 10-item CONSULT-PSYCHIATR scale in the independent confirmatory subsample.

Item	Standardized loading	R²
Item 1	0.7559	0.5714
Item 2	0.8784	0.7716
Item 3	0.8272	0.6843
Item 4	0.8697	0.7563
Item 5	0.9038	0.8169
Item 6	0.9104	0.8288
Item 7	0.8948	0.8007
Item 8	0.7837	0.6142
Item 9	0.8555	0.7319
Item 10	0.7351	0.5403

Confirmatory factor analysis was conducted in the independent confirmatory subsample (n = 1501). All standardized loadings were statistically significant at p < 0.001.

**Table 5:** Confirmatory model fit and reliability evidence for the 10-item CONSULT-PSYCHIATR scale.

Panel	Indicator	Value
A. Model fit of the 10-item one-factor CFA	**χ² (df)**	456.138 (35)
	p-value	< 0.001
	CFI	0.972
	TLI	0.964
	RMSEA (90% CI)	0.090 (0.082–0.097)
	SRMR	0.022
B. Reliability evidence	**Cronbach’s alpha, total sample**	0.9622
	Cronbach’s alpha, excluding constant-response patterns	0.9177
	Ordinal alpha, full polychoric EFA subsample	0.9737
	Ordinal omega total, full polychoric EFA subsample	0.9770
	Ordinal alpha, sensitivity subsample	0.9365
	Ordinal omega total, sensitivity subsample	0.9466

Panel A presents fit indices for the classical confirmatory factor analysis conducted in the independent confirmatory subsample (*n* = 1501). χ², chi-square statistic; df, degrees of freedom; CFI, Comparative Fit Index; TLI, Tucker–Lewis Index; RMSEA, Root Mean Square Error of Approximation; CI, confidence interval; SRMR, Standardized Root Mean Square Residual. Panel B summarizes classical and ordinal reliability estimates. Cronbach’s alpha was estimated in the total sample and after excluding constant-response patterns. Ordinal alpha and ordinal omega total were estimated from the polychoric exploratory factor analyses in the full exploratory subsample and in the corresponding sensitivity subsample excluding constant-response patterns.

Bold values indicate the main model-fit and reliability estimates highlighted for interpretation.

Because the items were ordinal, a complementary confirmatory analysis was conducted using generalized structural equation modeling with an ordered probit link. In this model, all non-constrained item loadings were statistically significant (*p* < 0.001), supporting the proposed common latent structure. The ordinal model yielded a log likelihood of −14765.391, with AIC = 29630.78 and BIC = 29896.48. A sensitivity ordinal model excluding constant-response patterns (n = 803) also converged and showed statistically significant non-constrained loadings, with a log likelihood of −9924.179, AIC = 19948.36, and BIC = 20182.78.

### 3.5 Reliability evidence and final CONSULT-PSYCHIATR scale

Reliability estimates are summarized in **Table 5B**. In the total sample, the 10-item CONSULT-PSYCHIATR scale showed very high classical internal consistency, with a Cronbach’s alpha of 0.9622. After excluding participants with constant-response patterns, Cronbach’s alpha remained high at 0.9177. Ordinal reliability estimates based on the exploratory polychoric matrices were also strong. In the full exploratory subsample, ordinal alpha was 0.9737 and ordinal total omega was 0.9770. In the sensitivity analysis excluding constant-response patterns, ordinal alpha was 0.9365 and ordinal total omega was 0.9466. Taken together, these findings indicate that the scale retained high internal consistency even under a more conservative analytic scenario.

Based on the content-validity process and the convergent exploratory, confirmatory, and reliability evidence, the final CONSULT-PSYCHIATR scale retained its 10 original items (**Table 6**). This decision prioritized preservation of the original construct coverage and avoided *post hoc* item deletion driven solely by model-fit optimization. The instrument is introduced by the stem: “I would not see a psychiatrist because…”, followed by five ordered response options ranging from Strongly disagree to Strongly agree. Higher total scores indicate greater endorsement of reasons for reluctance to attend psychiatric consultation.

**Table 6:** Final 10-item CONSULT-PSYCHIATR scale: original Spanish items and English rendering.

Item	Original Spanish item	English rendering
1	Porque atienden a personas con enfermedades mentales graves.	They treat people with serious mental illnesses.
2	Porque no creo que puedan entenderme.	I do not think they can understand me.
3	Porque no sé cómo expresar lo que me pasa y eso me frustra.	I do not know how to express what is happening to me, and that frustrates me.
4	Porque es para personas débiles.	It is for weak people.
5	Porque me van a juzgar.	They will judge me.
6	Porque es vergonzoso.	It is shameful.
7	Porque la terapia dura para siempre.	Therapy lasts forever.
8	Porque no tengo tiempo.	I do not have time.
9	Porque mis padres no me lo permiten.	My parents do not allow it.
10	Porque no soy violento/a.	I am not violent.

Instruction stem in Spanish: “No acudiría a consulta con un psiquiatra porque…”. English rendering: “I would not see a psychiatrist because…”. Response options in the original Spanish instrument: Totalmente en desacuerdo, En desacuerdo, Ni de acuerdo ni en desacuerdo, De acuerdo, Totalmente de acuerdo. Higher total scores indicate greater endorsement of reasons for reluctance to attend psychiatric consultation.

## 4 Discussion

The present study provides evidence of content validity, internal structure, and internal consistency for the CONSULT-PSYCHIATR, a brief instrument designed to assess reasons why workers in Peru may be reluctant to attend psychiatric consultation. The final version retained the 10 original items and showed convergent psychometric evidence supporting a predominantly unidimensional structure. This structure was observed in a polychoric exploratory factor analysis and subsequently examined in an independent confirmatory subsample, thereby strengthening the internal validation strategy of the study.

Although the observed factorial structure was unidimensional, the content of the 10 items reflects several closely related reasons for reluctance to seek psychiatric consultation: stigma-related beliefs, anticipated difficulties in the clinical interaction, and practical constraints. Several items address stereotyped or prejudicial beliefs about psychiatric care, including the idea that seeing a psychiatrist is only for people with severe mental illness, is shameful, or is a sign of weakness. These themes are consistent with evidence showing that stigma and negative attitudes toward mental illness and help-seeking can discourage service use and promote concealment, particularly when individuals anticipate judgment from others (5, 6). In occupational contexts, these concerns may be intensified by fear of labeling, discrimination, or adverse professional consequences associated with mental health disclosure (8, 17).

Second, the scale includes reasons related to anticipated difficulties in the clinical interaction, particularly not knowing how to express what is happening and doubting that the psychiatrist will understand. These expectations are relevant because communication and the perceived quality of the therapeutic relationship influence how acceptable, useful, and trustworthy professional care is perceived to be (18, 19). In this sense, reluctance to seek psychiatric care may arise not only from stigma, but also from doubts about whether the consultation will provide a respectful and meaningful interpersonal space.

Third, the item referring to lack of time captures a practical barrier that is particularly relevant in employed adults. This finding is consistent with the broader literature indicating that help-seeking is shaped not only by attitudes, but also by real-world access constraints. In workplace contexts, this point is especially important because interventions are unlikely to be effective if they rely only on awareness or screening without improving access pathways and conditions for care (9, 10). Thus, a brief instrument focused on concrete reasons for reluctance may help occupational health services and workplace wellbeing programs move beyond generic messaging and identify modifiable barriers that can be addressed through clearer information, facilitated referral pathways, or protected time for appointments.

From a measurement perspective, the findings support interpreting the scale as a single summary measure of reluctance-related reasons rather than as a set of separate subscales. In the exploratory subsample, the first factor was dominant and accounted for a large proportion of the variance, while all factor loadings were high. In the independent confirmatory subsample, the one-factor model also showed strong standardized loadings and substantial item-level explained variance. This pattern suggests that the items behave as closely related indicators of a broader latent construct of reluctance to attend psychiatric consultation. Such a structure may be advantageous for brief assessment in occupational or public health settings, especially when the goal is to summarize the overall intensity of perceived barriers rather than to classify individuals into distinct dimensions.

A notable feature of the dataset was the high proportion of participants who selected the same response category across all 10 items. Rather than treating this pattern as analytically negligible, the study quantified its frequency and incorporated sensitivity analyses excluding these participants. The persistence of the unidimensional structure and the maintenance of high reliability estimates after this exclusion provide additional support for the robustness of the main conclusions. At the same time, the reduction in factor loadings and explained variance in the sensitivity analysis indicates that the magnitude of the psychometric estimates in the full sample should be interpreted with appropriate caution, as highly homogeneous response behavior may have contributed to the strength of the observed associations.

The confirmatory findings also warrant balanced interpretation. The classical one-factor model showed favorable CFI, TLI, and SRMR values, together with high standardized loadings across all items. However, the RMSEA was less favorable. Rather than using modification indices to drive *post hoc* item deletion or model re-specification, the model was retained in its theoretically grounded 10-item form and interpreted in light of the full body of evidence, including the exploratory polychoric analysis, the independent confirmatory evaluation, the ordinal confirmatory model, and the sensitivity analyses. This analytic choice preserves the content coverage of the instrument while avoiding overfitting to a single sample.

The study also contributes to the measurement of mental health help-seeking barriers in the Peruvian context. Peru has expanded community-based mental health services, yet greater availability of care does not necessarily eliminate reluctance to seek psychiatric consultation. In this setting, the CONSULT-PSYCHIATR may be useful not as a diagnostic tool, but as a pragmatic instrument for identifying perceived reasons for avoiding psychiatric care among workers and informing targeted strategies in occupational or organizational settings (11, 12). Compared with broader instruments assessing self-stigma, general barriers to care, or attitudes toward professional psychological help, this scale focuses specifically on concrete reasons for reluctance to attend psychiatric consultation in a working population, which may enhance its practical interpretability in this context (13–15).

### 4.1 Limitations and future directions

The findings should be interpreted in light of several limitations. First, the instrument was evaluated in employed adults from selected departments of Peru; therefore, generalization to workers in other geographic, occupational, or sociocultural settings, as well as to rural communities, adolescents, unemployed individuals, or clinical populations, should be made with caution. Second, participants were recruited through non-random procedures, which may limit representativeness and introduce selection bias. Third, a considerable proportion of participants showed constant-response patterns across all items. Although sensitivity analyses supported the stability of the main psychometric conclusions, this response pattern may reflect uniform endorsement, acquiescent responding, low engagement, or a combination of these mechanisms, which could not be fully disentangled in the present study. Fourth, the confirmatory one-factor model yielded mixed global-fit evidence, with favorable incremental and residual-based indices but a less favorable RMSEA; consequently, the proposed structure should be further evaluated in independent external samples. Finally, the present study focused on content validity, internal structure, and internal consistency. Future research should examine test–retest reliability and additional sources of validity evidence, including convergent, discriminant, and criterion-related validity, for example through associations with self-stigma, broader barriers to care, attitudes toward professional help-seeking, help-seeking intentions, or actual service use.

## 5 Conclusion

The CONSULT-PSYCHIATR is a brief instrument with evidence of content validity, internal structure, and internal consistency for assessing perceived reasons why workers in Peru may be reluctant to attend psychiatric consultation. The scale may be useful for occupational health research and workplace mental health initiatives aimed at identifying modifiable barriers to care. Nevertheless, these findings should be considered preliminary, and additional studies are needed to confirm the performance of the instrument in other populations, settings, and external validity criteria.

## Data Availability

The original contributions presented in the study are included in the article/**Supplementary Material**. Further inquiries can be directed to the corresponding author.

## Supplementary material

Supplementary Table 1 Summary of constant-response patterns in the CONSULT-PSYCHIATR scale.

Supplementary Table 2 Absolute and relative response frequencies by item and response category.
