# Supplementary material for: Validation of the CONSULT-PSYCHIATR Scale: reasons for reluctance to seek psychiatric consultation among workers in Peru
Source: Front Psychiatry. 2026 Jul 8;17:1836597. doi: 10.3389/fpsyt.2026.1836597 (PMC13388752; doi:10.3389/fpsyt.2026.1836597)
Supplement: Supplementary Table 1 — Summary of constant-response patterns in the CONSULT-PSYCHIATR scale. [file DataSheet1.docx]

**Supplementary Tables**

| **Supplementary Table S1. Summary of constant-response patterns in the CONSULT-PSYCHIATR scale** | | |
| --- | --- | --- |
| **Indicator** | **n** | **%** |
| Total sample | 3001 | 100.00 |
| Participants with constant response across the 10 items | 1409 | 46.95 |
| Participants without constant response across the 10 items | 1592 | 53.05 |
| Note. Constant response was defined as selecting the same response category across all 10 items. | | |

| **Supplementary Table S2. Absolute and relative response frequencies by item and response category** | | | | | |
| --- | --- | --- | --- | --- | --- |
| **Item** | **0 n (%)** | **1 n (%)** | **2 n (%)** | **3 n (%)** | **4 n (%)** |
| Item 1 | 603 (20.09) | 784 (26.12) | 830 (27.66) | 575 (19.16) | 209 (6.96) |
| Item 2 | 623 (20.76) | 915 (30.49) | 943 (31.42) | 407 (13.56) | 113 (3.77) |
| Item 3 | 577 (19.23) | 846 (28.19) | 914 (30.46) | 520 (17.33) | 144 (4.80) |
| Item 4 | 744 (24.79) | 882 (29.39) | 931 (31.02) | 344 (11.46) | 100 (3.33) |
| Item 5 | 687 (22.89) | 904 (30.12) | 925 (30.82) | 389 (12.96) | 96 (3.20) |
| Item 6 | 662 (22.06) | 889 (29.62) | 902 (30.06) | 442 (14.73) | 106 (3.53) |
| Item 7 | 666 (22.19) | 918 (30.59) | 912 (30.39) | 399 (13.30) | 106 (3.53) |
| Item 8 | 538 (17.93) | 831 (27.69) | 917 (30.56) | 544 (18.13) | 171 (5.70) |
| Item 9 | 747 (24.89) | 914 (30.46) | 938 (31.26) | 310 (10.33) | 92 (3.07) |
| Item 10 | 652 (21.73) | 772 (25.72) | 933 (31.09) | 453 (15.09) | 191 (6.36) |
| Note. Values are absolute frequencies with percentages in parentheses. N = 3001 for each item. | | | | | |
